# Mechanical Properties and Seismic Performance of Wood-Concrete Composite Blocks for Building Construction

**DOI:** 10.3390/ma12091500

**Published:** 2019-05-08

**Authors:** David Dominguez-Santos, Daniel Mora-Melia, Gonzalo Pincheira-Orellana, Pablo Ballesteros-Pérez, Cesar Retamal-Bravo

**Affiliations:** 1 Department of Engineering and Building Management, University of Talca, Curicó 3340000, Chile; ddominguez@utalca.cl (D.D.-S.); ceretamal@utalca.cl (C.R.-B.); 2 Department of Industrial Technologies, University of Talca, Curicó 3340000, Chile; gpincheira@utalca.cl; 3 Department of Mechanical Engineering and Industrial Design, University of Cadiz, 11519 Cadiz, Spain; pablo.ballesteros@uca.es

**Keywords:** seismic-resistant behavior, materials, structures, concrete, sawdust, wood shavings

## Abstract

Recent catastrophes that occurred during seismic events suggest the importance of developing new seismic-resistant materials for use in building construction. Ordinary concrete is one of the most common materials in buildings. However, due to its low ductility and flexural strength, its seismic behavior can be improved upon by different additives. In this regard, wood-concrete composites exhibit desirable structural properties not achievable by either wood or concrete alone, making it an interesting material from a seismic point of view. This work analyzes and compares the performance of blocks built with ordinary concrete versus blocks built using different wood additives (sawdust and wood shavings). This includes the construction of concrete blocks in a lab, determination of their construction and seismic-resistant properties, as well as an analysis of their performance in buildings with a different number of storeys. The results show how blocks with wood aggregates comply with current regulations for structural materials in a seismic country like Chile, while also considerably outperforming traditional concrete blocks in the event of an earthquake.

## 1. Introduction

Recent figures confirm that buildings represent approximately 32% of the world energy consumption and that their construction generates almost 20% of the greenhouse gas emissions [1]. In this regard, globalization and the growing scarcity of resources makes it necessary to develop more sustainable materials and to improve those that already exist, keeping construction costs as low as possible.

In recent years, various catastrophes caused by earthquakes have occurred in countries, such as Haiti, Chile, and Japan. These phenomena show that how we build and the materials we use are key elements, even in countries with earthquakes of low or moderate magnitude [2]. Therefore, the use of composite materials that decrease the weight of the structure, while improving the ductile behavior of the building may be advisable, since the building would have better seismic behavior. 

Due to its versatility and easy assembly [3], concrete is one of the most used building materials, both in structural and non-structural elements. Concrete is malleable and can adopt almost any shape. It also has a high compressive strength and is steel adherent. In fact, due to the latter, concrete is also capable of increasing its tensile strength and ductility, something particularly relevant during seismic events.

In its most basic definition, concrete is a material formed by a binder—usually cement [4,5]—to which aggregates and water in varying proportions are added. However, it is also possible to add other minor components and/or specific additives to modify some of their properties. 

The main contribution of this work is to study whether the addition of wood to cement allows the manufacturing of concrete blocks with enhanced seismic behavior, thus, without seriously altering other resistant properties and increasing its manufacturing costs. Furthermore, the analysis is extended to analyze and compare the performance of blocks with ordinary concrete and blocks with wood additives in compression and flexural tests. Finally, the performance of these blocks in buildings of different stories is compared as well.

Ordinary concrete blocks as a construction element are ubiquitous in building construction [6,7,8]. Unfortunately, the behavior of these elements during earthquakes [9], as well as the construction processes [10] and the sustainability of their manufacture are clearly improvable [11]. These limitations, along with an absence of studies on the addition of wood-based materials and their effect on seismic-resistant properties in concrete blocks justify this work.

The remainder of the paper is organized as follows. The *Background* section reviews the major contributions published on the topic. The *Materials and Methods* section shows the construction procedure adopted for the production of the concrete blocks. The *Mechanic Characterization of the Concrete Blocks* section describes the characterization of the blocks and all the tests that were carried out on them. It also describes the mechanical properties that could improve their seismic behavior on adding wood, such as improved ductility or flexural strength. Next, the *Structural Analysis of Concrete Blocks with Wood Additives* section describes the structure modeling and seismic behavior of some representative frames. The buildings’ performance considering static and dynamic nonlinear analysis are analysed and reported in the *Buildings Performance* section. The *Discussion* section provides insights and further analysis of the implications of the results. Finally, the *Conclusions* summarizes the paper and makes suggestions for future works.

## 2. Background 

Mortar and cement concretes often contain chemical or mineral additives. The purpose and dosage of these additives are to modify one or more properties of the mixture, improving its performance either in their fresh or hardened state. Similarly, it is to be expected that these additives do not excessively and negatively worsen other mechanical characteristics of the mixture, nor reduce its durability or accelerate the corrosion of its embedded steel elements.

In the scientific literature, there are abundant studies on the addition of endless additives to the traditional concrete mixture. The addition of metal or polypropylene fibers to the mixture, for example, increases properties such as load capacity [12,13,14], shear strength or ductility [15]. Likewise, but from an environmental point of view (damage caused by the raw materials extraction and/or the CO_2_ emissions), some mixtures have considered the addition of volcanic ashes [16], wood-based materials [17], recycled concrete [18,19,20], and many others. 

Concerning concrete blocks, these are frequently used by many countries in enclosures (structural and non-structural) and in room-dividing elements due to their low cost and good construction characteristics. Indeed, but only recently, numerous researchers have studied the influence of different additives on the thermal [21,22], acoustic [23] and mechanical [24] properties of such concrete blocks. 

Among the different materials that can be used as an additive, wood has interesting properties from a seismic point of view [25,26,27]. It performs well when bent and compressed [3], and it also has low thermal [28], acoustic and electrical conductivity [29,30,31]. It worth noting that seismic countries such as Chile, Colombia and others in Southeast Asia have large reserves of wood [32,33]. These countries also process this material to create multiple derivative products. This means they also have high volumes of wood waste in factories and sawmills that can be potentially used for other purposes [34].

Some research has also added wood fibers to cement [22,35], where the two main disadvantages are the high moisture absorption and low compatibility between fibers and cement [36]. Additionally, all these studies conclude that the addition of wood decreases compressive strength, but none of them has analyzed the potential flexural strength increase in concrete blocks, nor their seismic-resistant properties. The present study takes a first step in this direction and tries to understand how they can perform in structures with multi stories.

## 3. Materials and Methods

### 3.1. Considerations and Previous Calculations

It is evident that the additives dosage in concrete mixtures determines its final properties (e.g., strength, ductility, workability). In this work, the methodology used for the manufacturing of the blocks followed the Chilean standard for concrete and aggregates [37,38]. For those readers that are not familiar with this standard, its essential aspects are described as supplemental material. Nonetheless and without going into much detail, the Chilean standard has a high level of resemblance with most concrete regulations from European countries.

The Chilean standard particularly proposes to carry out different granulometry tests for the fine and coarse aggregates that will be used in the fabrication of concrete. After the granulometry tests, different properties such as density, humidity and water absorption are measured. Other important properties in the characterization of the mixture are dry bulk density, compacted bulk density, hollow content and foaming. Table 1 shows the key property values of sample concrete prepared in laboratories at the University of Talca (Chile), whose facilities were used for this study.

### 3.2. Construction of the Blocks

**Figure 1:**
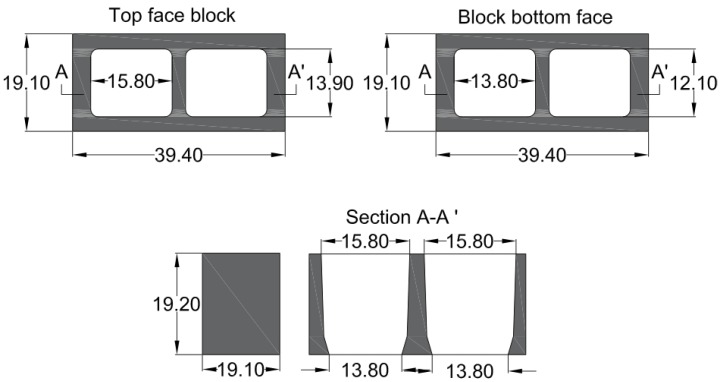
Dimensions (cm) of concrete blocks used in the tests.

Manufacturing of the concrete followed the recommendations of Chilean standards, which also comply with US [39,40] and Spanish standards [41,42]. These standards specify the dosages of water, cement, gravel, and sand for the preparation of one m^3^ of concrete suitable for structural use. The geometric dimensions of the constructed blocks are shown in Figure 1. These dimensions are suitable for structural use and buildings for commercial use [43]. The different tests and samples of concrete blocks carried out also considered Chilean NCh1019 [44] and NCh1037 [45] standards. 

**Figure 2:**
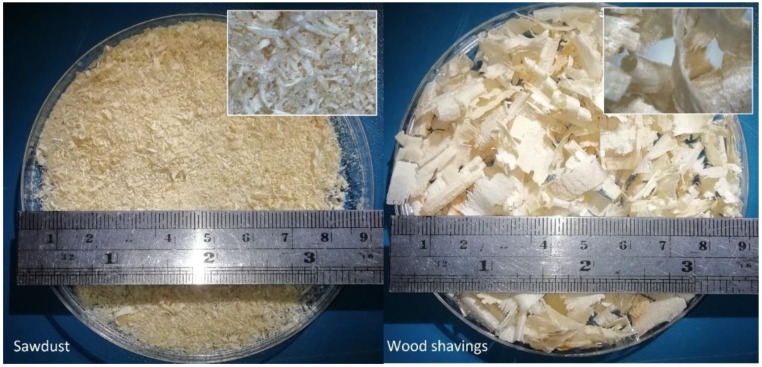
Left: Sawdust additive. Right: Shavings additive.

The mixtures used for manufacturing of the blocks in this study replaced part of the gravel by wood additives (sawdust or wood shavings). More precisely, four different percentages of additive (10%, 15%, 25% and 40% of gravel weight) were considered in the mixture preparation. Figure 2 presents the two wood additives used in the concrete, with large dimensional differences between them. On one hand, sawdust shows an average particle size of 1 mm^2^, with 80% of the material between 0.5 and 1.5 mm (with sieving). On the other hand, wood shavings show an average of 1 cm^2^ without sieving. However, despite the difference, the zoomed version of each image shows that the wood does not lose its fibrous structure. 

It should be noted that when adding wood to the mixture, the density decreases compared to that of the traditional concrete (approx. 2425 kg/m^3^). The seismic actions induce inertia forces that are proportional to the building mass. Consequently, lower weights in the structures will provide an improvement in the anti-seismic behavior of the building under the same structural conditions. 

Considering the water absorption and particle distance with each other as a consequence of the existence of water when the latter evaporates, the concrete density also varies. Among the concretes tested, the water absorption in mixtures with sawdust was found to be significantly higher. Hence, these concretes turned out to be the lightest. The differential water absorption also allows understanding of the volumetric expansion observed in concretes with wood additives (wood shavings and sawdust).

Based on the standards and additives considered for each case study, Table 2 shows in percentage the mass, the proportions of each material used per m^3^ of concrete mixture, as well as their densities.

Once the dosages were determined, the blocks were manufactured using a metal plate formwork, whose geometric details can be found as supplemental material. After filling the form, the mixture was left to rest for a few minutes to achieve a homogeneous texture. The pouring process was carried out by layers (between three and five concrete layers per block), adequately vibrating the mixture to eliminate the openings caused by the air. Hours later, the blocks were unmolded and immersed in water for 28 days for correct curing and subsequent homogeneous drying. After curing and drying, all mechanical strength tests were carried out. 

## 4. Mechanic Characterization of the Concrete Blocks

This section analyzes the properties of the concrete blocks manufactured using wood shavings and sawdust additives. For each type of block described in Table 2, five samples were fabricated. Later, we will only show the average results of each set of five samples. 

The blocks were subject to compression and four point flexural tests according to the Chilean standard NCh 181 [43]. In particular, for each sample, its mechanical capacity was determined while also recording its compression and flexural capacity curves.

**Figure 3:**
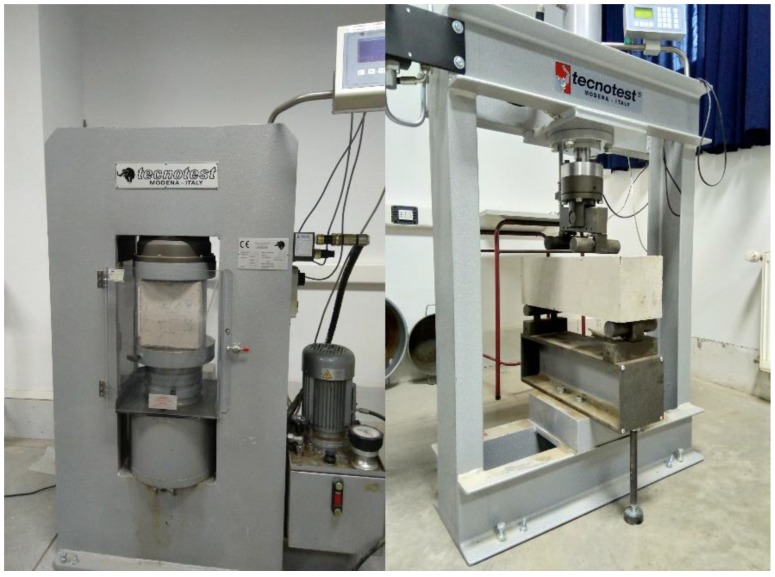
Blocks compression and flexural tests.

Compression and flexural tests were carried out using hydraulic machines (Figure 3). In the case of compression tests, the measurement protocol requires an even distribution of loads on the face of the block to avoid measurement errors a consequence of possible surface irregularities. Hence, all blocks were introduced inside the machine (Figure 3 left side) after having ensured that the upper and lower sides were parallel by means of a plastic mortar layer (3 mm thick) and by using 10 mm-thick metal plates. 

In the case of the flexural tests, the protocol began by marking the central point in the blocks. From this point, two marks were made at 7.5 cm from the center. These marks correspond to the points of support and where the loads were applied (Figure 3 right side).

### 4.1. Compressive Strength

**Figure 4:**
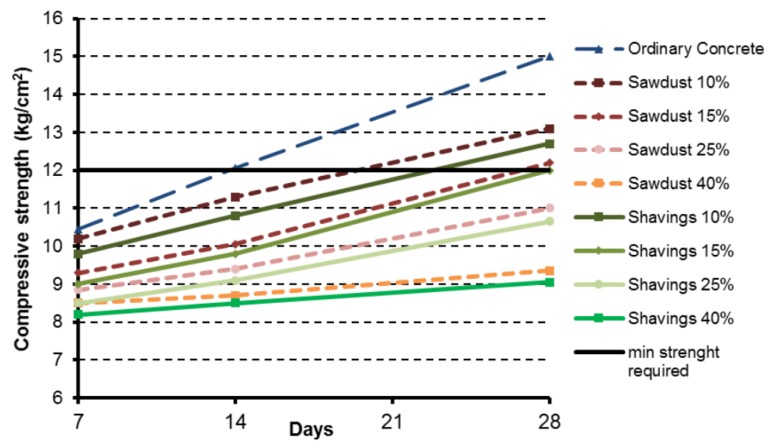
Daily evolution of the concrete block compressive strength.

The average compressive strength of all concrete blocks was measured at 7, 14 and 28 days (Figure 4). As expected, the highest compressive strength corresponded to those samples made of ordinary concrete. Regarding the concretes with wood additives, the samples increased their compressive strength significantly as the mixture dried. Nevertheless, Figure 4 shows for wood-composite concretes a lower increase rate compared to ordinary concrete. It should be noted that the minimum compressive strength for a concrete block to be used as a structural element according to the Chilean standard NCh 181 (2006) is 12 kg/cm^2^. As a result, samples with 25% and 40% of wood additives had to be excluded from the rest of the analyses, as their compressive strength was insufficient. 

On comparing the two types of wood additive, the concrete blocks with sawdust recorded a higher compressive strength than those with wood shavings. The compressive strength of ordinary concretes at day 28 was 22.2% and 27.9% higher than blocks containing 10% of sawdust or wood shavings, respectively. As expected, the difference in compressive strength between ordinary concrete and wood-based concrete increased as the proportion of sawdust or wood shavings increased. 

### 4.2. Flexural Strength

As a consequence of the previous non-conformity of concrete blocks with 25% and 40% of wood additives, the flexural tests also considered the concretes with up to 15% of sawdust or wood shavings. Table 3 shows that the average flexural strength observed 28 days after the mixture was manufactured. At day 28, it is generally accepted that the concrete strength is close to its maximum.

The results show how concrete blocks with sawdust or wood shavings increase up to 10% the flexural strength compared to traditional concrete. Also, the flexural strength between concretes with sawdust or wood shaving hardly show any difference (the former is just 3% more resistant than the latter).

### 4.3. Capacity Curves (Compressive Force—Deformation) and Ductility (μ) of Compression Tests

**Figure 5:**
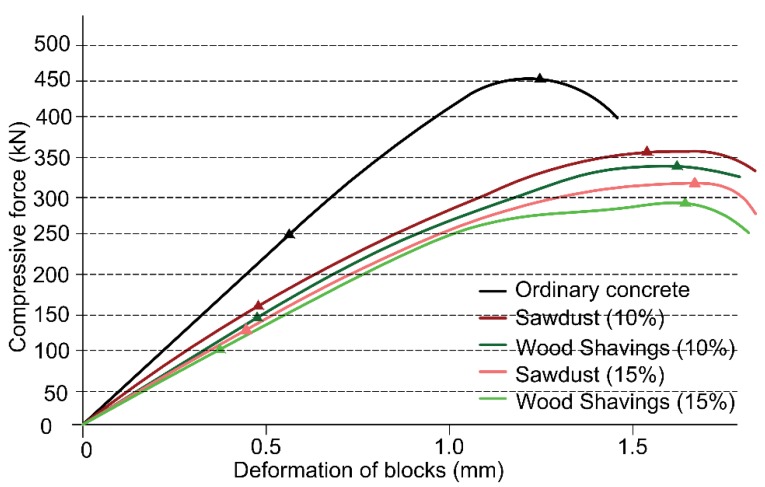
Capacity Curves (Compressive Force—Deformation) of the Concrete Blocks Suitable for Structural Use.

Figure 5 shows the compressive capacity curves (force-deformation) of concrete blocks 28 days after their fabrication. Not being suitable for structural use, blocks with additives of more than 15% wood were not considered either. The capacity curves are obtained according to Chilean standard NCh 1037. This test is performed at a constant load of 0,25 MPa/s ± 0.05 MPa/s until break. 

As expected, the results show that the ordinary concrete blocks are more resistant than the wood-based blocks, but also less ductile. Both properties are very important in seismic-resistant structural design. On one hand, ordinary blocks are approximately 20% more resistant than blocks with 10% wood additives. This difference increases up to 35% when wood additives reach 15%. Additionally, a decrease in block stiffness can be noticed in the elastic zone of the capacity curve, with reductions of 25.1% (10% sawdust); 28.2% (10% shaving); 31.9% (15% sawdust), and 40.5% (15% shaving) respectively. The fact is that this was expected due to the low mechanical properties of the wood. On the other hand, ductility increased considerably. In this case, ductility was obtained from the yielding point of each block according to the criteria established in ATC-40 [46] and FEMA P-1050-1 [47]. Table 4 shows the limits of plastic deformation (*δ_p_*), the ultimate deformation (*δ_u_*) and ductility obtained as the ratio between both deformations (*μ* = *δ_u_*/*δ_p_*), for all blocks tested. Specifically, the increases registered were 44.5% (10% sawdust); 55.1% (10% shaving); 75.3% (15% sawdust) and 95.2% (15% shaving), respectively. It is important to highlight that ductility is very important in seismic-resistant structural design, having better seismic-resistant properties with greater ductility.

As a comparison between both additives, sawdust blocks seem slightly more resistant at compressive forces than wood shaving blocks, but not in terms of ductility, where it is possible to see a greater influence of wood-shaving comparing to sawdust. The results summarized in Table 4 show how concrete blocks with wood additive are more ductile than ordinary concrete; the blocks with wood shavings were the most ductile.

These results offer interesting applications in structures exposed to high and/or moderate seismicity. However, it is also necessary to analyze the way blocks are structurally connected with each together and the structure itself in order to observe to what extent this additional ductility can be exploited. This is the objective of the following section.

## 5. Structural Analysis of Concrete Blocks with Wood Additives

This section describes the modeling and seismic behavior of some representative building frames using nonlinear static and dynamic analyses. Calculations were implemented with the structural analysis software Seismostruct v.7.0.2. This software is based on finite elements and is a product of the company Seismosoft^®^ (Pavia, Italy) [48]. This software allows to estimate the displacement in space frames under static and dynamic loads considering the behavior of nonlinear materials across all their geometries.

For a representative analysis of the performance of the different concrete blocks, the maximum displacements and maximum strengths of capacity curves at the top floor of different buildings were evaluated. Particularly, capacity curves were compared using non-linear analyses (push-over), whereas maximum displacements were measured by non-linear dynamic analysis. For the latter analyses, a register of a medium-to-moderate intensity earthquake was used, (described later in the paper).

### 5.1. Description of the Frames Analysed

The structures analysed in this work focus on frames due to the abundance of this construction system in many Latin American and European countries [49,50]. These structures are characterized by fast execution times and the need for relatively few resources. Both aspects are directly related to the reduction of construction costs. 

Traditionally, the structural behavior of frames made exclusively of beams and columns during earthquakes has not been satisfactory. Consequently, it is advisable to incorporate walls with concrete blocks to mitigate possible damage [49,50]. In this study, the structural behavior of frames with 2 and 5 stories has been analysed.

In recent years, most European countries have been adapting their national (structural) codes to resemble European codes (Eurocodes). For this reason, the Eurocode of structural design [51] was considered for the structural design of the frames presented later. Consequently, the results of this work can be considered representative of a significant percentage of existing buildings in Europe and part of Latin America.

**Figure 6:**
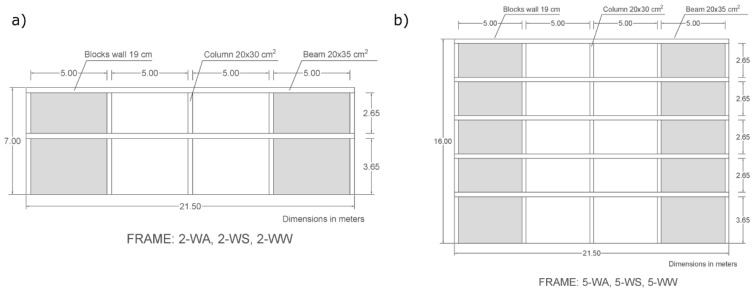
Frames considered in the analysis: (**a**) 2-storey frame; (**b**) 5-storey frame.

As shown in Figure 6a,b, the frames in elevation are formed by four bays five meters wide each. Two of these bays are assumed to be filled with concrete block walls, whereas the other two are left empty. The windows of the building were located in the latter. 

**Figure 7:**
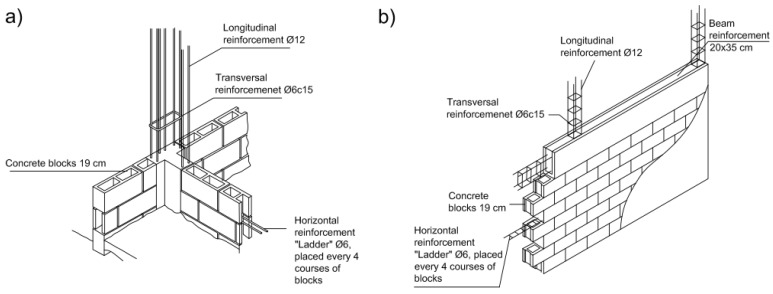
Arrangement of blocks. (**a**) assembly I; (**b**) assembly II.

The blocks and openings in the frame bays are located from the beam below to the top beam without any anchor to the primary structural elements apart from the reinforcements, which are placed every four lines of blocks and anchored to the columns (Figure 7a,b).

The height of each storey is three meters, except for the ground floor, whose height is four meters. This means the buildings were 7 and 16 m high (for the 2- and 5-storey frames), respectively. The frames’ configuration is regular and symmetrical in elevation. The frames analysed correspond to the external façades of the buildings.

Concerning how the block corners and intersections were assembled, the vertical reinforcements are placed across the blocks, filling th**e** openings with concrete. The vertical reinforcement is also tied transversely to the stirrup. On the other hand, every four lines of blocks, a transversal reinforcement is placed on the blocks and tied transversely to the frame columns. Figure 7a,b show the specific arrangement of the blocks and reinforcements in the frames.

The horizontal structural elements that join the frame bays correspond to 20 cm wide and 35 cm high beams in all the stories. The vertical elements are made up of five 20 × 35 cm columns. Structural continuity is achieved through the reinforcements (the steel rods connecting the beams and columns), as well as the horizontal reinforcements between the blocks and columns. This layout meets all requirements of the European structural design codes.

The non-structural walls are made of 19 cm-thick blocks separated by 1 cm-thick mortar and with transversal steel ladder-type reinforcements every four lines of blocks (Figure 7a,b).

The most important characteristics and properties of the models used in this study for seismic behavior purposes are summarized in Table 5. The materials used for the main structural elements (beams and columns) were concrete type HA-25 and steel B-500-S. Both materials are completely defined in CTE DEB SE AE standard [52]. 

In Table 5, each frame is denoted by a number and two letters. The first number indicates the amount of building stories and the letters the type of block used in the walls: “WA” stand for blocks with no aggregates, “WS” means blocks with sawdust aggregates, and “WW” means blocks with wood shaving aggregates. 

Table 5 also shows the configuration of the structural elements of each storey, the weight of each frame following the G + 0.3Q approach according to Eurocode 8 [53], and the fundamental periods of the frames using the different configurations of blocks.

The initial characteristic strength of the structural concrete was assumed as f_ck_ = 250 kg/cm^2^, and the steel yield strength f_yk_ = 5.000 kg/cm^2^ (as expected for a steel type B 500-S). 

Given its high fragility, the present work did not account for the potential collaboration of window carpentry in the building openings. Finally, because the analysis is based on frames of residential buildings, administrative buildings, and small stores, live loads of 2 kN/m^2^ [52] were considered in all stories, except for the top floor (roof). In the roof, a load of 1 kN/m^2^ was assumed.

### 5.2. Representative Earthquake

The earthquake in Lorca (Spain) that occurred in 2011 has been chosen as representative medium intensity earthquake for the building performance analyses shown later. After the occurrence of this earthquake, seismic vulnerability studies became more important in some European countries with similar building structures. These countries included Spain [54,55], France [56,57], and Italy [58].

According to the Geological and Mining Institute of Spain, the Lorca earthquake was one of the most destructive earthquakes registered in Spain, despite its moderate magnitude (M_w_ = 5.1). Further details of this earthquake exceed the scope of this paper, but a complete description along with its accelerogram and response spectra can be found in [59].

### 5.3. Main Frame Modelling

The main frame was modeled using finite bar elements [60,61]. Each structural element (columns and beams) was specified individually following the prescriptions proposed by Mander for concrete [62] and the bilinear model of Ferrara [63] for reinforced steel bars.

In particular, the existing beams were represented by non-linear finite bar elements [64], where the non-linearities were concentrated in the plastic hinges located at the ends, equivalent to 15% of the total length of the element [60]. According to Scott et al. [65], the joints/connections between the wall blocks and the concrete beams were assumed to be rigid, while the hysteretic behavior representing the stress distribution was modeled by fiber models based on the properties of the material and the shape of the structural elements (each element section was discretized with 300 fibers). It was considered that the loads were applied on beams or linear horizontal elements.

The tolerances used for the displacement and rotations were of the order of 10^−5^ in both cases. A maximum number of 300 iterations was deemed sufficient.

For the numerical analysis, the Newmark-β method was used [66]. The Newmark method can be considered a generalization of the linear acceleration method. It is a numerical integration method widely used in the numerical evaluation of the dynamic response of structures. In this method, Beta (β) and Gama (γ) factors are coefficients that depend on the natural frequency (w) and damping (Ϛ) of the structure. For this work, β = 0.25 and γ = 0.5 were used, since with these values the Newmark-β method is implicit and unconditionally stable [67]. Finally, the Rayleigh model of 4% (mode 1) and 6% (mode 2) was considered [68]. The method for the modal combination used in the analyses was the complete quadratic combination (CQC) with a damping corresponding to 0.04.

The structural results included maximum shear at the base of the frames, seismic forces of each storey and maximum frame deformations taking into account both elastic and plastic structural behaviors. To obtain these results, different performance criteria were defined. The simulation of the behavior of each material in the frame elements requires entering data. Thus, the experimental values of plasticization and breakage are obtained from the capacity curves (Figure 5), while the unit deformations corresponding to the concrete and steel failure processes use the standard Seismostruct values [69,70,71]: concrete cracking (0.0001), spalling of concrete cover (−0.002), crushing concrete core (−0.002), creep (0.0025) and Steel fracture (0.06). Furthermore, the criteria referring to the curvature and rotations verified the rotation capacity of Mergos and Kappos [72] and the shear capacity established on the EC 8 Eurocode.

For this structural analysis, a 2D model was used due to the symmetry in each direction and to decrease the computational calculation time. These two-dimensional analyses were compared with three-dimensional analyses later in some cases, obtaining very similar results.

### 5.4. Infill Wall Modelling (Concrete Blocks Walls)

The presence of infill panels considerably modifies the structural behavior of RC structures. The infill wall modelling must consider the nonlinear inelastic behavior, the determination of mechanical properties and the interaction with the frame. However, there are many techniques that allow analysis of infilled frames. In this regard, Crisafulli et al. [73] made a detailed review of several publications on this subject. After conducting a literature review, this work adopts the double strut approach proposed by Crisafulli [74] and implemented by Piestley et al. [75] using software Seismosoft^®^. The main criteria considered for selection of the model was that this approach provides a good insight into the panel-frame interaction effects at a reasonable modelling and computational cost. Additionally, the double strut approach has been successfully applied for the prediction of the seismic response of multi-storey infilled reinforced concrete frames, with verified results [76]. 

**Figure 8:**
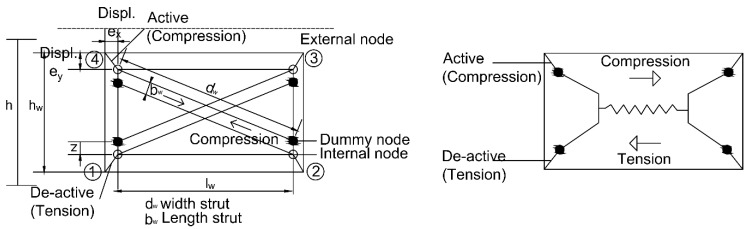
Infill wall model.

The approach of Crisafulli proposes a macro-model for the evaluation of the global response of this system. The model is implemented as a four-node panel element, which is connected to the frame at the beam-column joints (Figure 8). Internally, the panel element accounts separately for the compressive and shear behavior of the masonry panel using two parallel struts and a shear spring in each direction. This model allows to properly consider the lateral stiffness of the panel and the strength of masonry panel, particularly when a shear failure along mortar joints or diagonal tension failure is expected. Further numerical details on the transformation of the forces at the internal and dummy nodes to the external forces at the four nodes can be followed in [76].

For model calibration in terms of block walls, some geometrical and mechanical parameters are required to define the behavior of the masonry strut. A complete definition of each parameter can be found in [74]. For easier reference, all parameters obtained in the laboratory tests directly or indirectly are presented in Table 6. In addition, Table 7 shows the numerical values for these parameters for each type of concrete. 

On the other hand, Table 8 presents parameters whose values have been assumed from a literature review. It should be noted that in this case, these values have been validated in previous works. Additionally, the assumed values are the same for ordinary concretes and those with additives.

Finally, in addition to geometric and mechanic parameters, a set of empirical factors associated exclusively with the cyclic rules need also to be defined in the model. Table 9 shows the suggested values obtained by [74] after calibration of experimental data and selected values for this work. A short explanation about the meaning of these parameters can be found in [79].

## 6. Buildings Performance

Different structural design codes specify different procedures for buildings seismic analysis. This paper considered both non-linear static analysis (push-over) and dynamic analysis, which are described in this section.

These analyses are carried out on the structures defined in the previous section, built with both ordinary concrete blocks and concrete blocks with up to 15% added wood shavings or sawdust. Calculations with these additives’ proportion were already justified by observing how compression tests ruled out samples with higher contents of sawdust and/or wood shavings as not being suitable for structural elements. 

### 6.1. Non-Linear Static (Push-Over) Analysis

Non-linear analyses (push-over) allow calculation of the maximum horizontal strength capacity of structures whose dynamic response is not significantly affected by the levels of deformation experienced. That is, the distribution of horizontal forces that simulate the dynamic response can be assumed constant. Non-linear static analysis is one of four analysis procedures embodied in FEMA 356/ASCE 41 and is commonly used in performance-based design approaches. For interested readers, a complete description of the method can be found in [49,69]. 

The methodology followed in this work concentrates the failures of the frames in the plastic hinges that appear in the areas near the nodes of each structural element (beams and columns). The analyses were made assuming triangular load distributions. This load pattern is increased proportionally with a factor (*λ·p*) until structural instability is reached. Additionally, a response control corresponding to an increase in the top floor nodes displacement is used.

**Figure 9:**
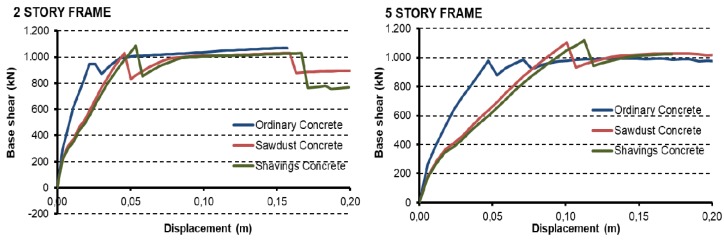
Capacity curves: (**left**) 2-storey frame; (**right**) 5-storey frame.

Figure 9a,b show the frames’ capacity or “push” curves described in Table 4 using H-25 concrete (25 MPa) and B-500-S corrugated reinforced steel (500 MPa), using ordinary blocks and blocks with 15% sawdust or wood shavings additives. This allows us to evaluate the influence of the increase in ductility and reduction of compression strength in general structural behavior.

Figure 9 shows the frames’ “nonlinear” static behaviour. The left part of both figures allows to see the elasto-plastic behavior of frames before a major failure. The beginning of each curve allows to see the elasto-plastic behaviour of the structures before a major failure. In the elastic zone, small differences were appreciated between structures built with ordinary concrete and structures built with wood-based concretes. Nevertheless, as the applied force increases, the displacement differences are greater for the same load level. During the load increase from 0 kN to the first major failure (approximately at 1000 kN), the structures support the shear forces with structural elements (such as beams and columns) and with concrete walls. These last elements increase the initial stiffness of the structure, but they fail earlier than the main frame and, consequently, a reduction in the structural stiffness can be observed up to the major failure. 

The plasticity in the capacity curves is generated mainly by cracking in the concrete and plasticity of steel reinforcement. These effects are represented in the model by the formation of plastic hinges in each element, and the length of these curves depends on the number of hinges generated prior to becoming an unstable structure. In the plastic propagation, the behavior of the structures is very similar for all cases, because they are being supported almost exclusively by the frames without walls. Regarding the structural performance of frames built with wood-composite blocks, it is possible to see an increase in the maximum force supported comparing with ordinary concrete structure before a major failure for the 5-storey frames. The maximum reached value is approximately 1100 kN in both materials, 10.7% higher compared to ordinary concrete (994 kN). After these important failures, a slight decrease in the structural performance can be observed for the 2-storey frame case, but a remarkable improvement in global behavior of the structure can be noticed in the 5-storey frames. This could be associated with the increase of ductility to the material due to wood additives and the effects could be more evident in higher frames.

Finally, due to the increase in ductility it is possible to see that the first major failure in the 2-storey frame occurs at 53 mm displacement in the frames with wood shavings blocks, at 45 mm in the frames with sawdust blocks and at 25 mm in the frames with ordinary concrete blocks. Similarly, for 5-storey frames, the first important failure occurred at a displacement of 11.5 mm in the frames with wood shavings blocks, at 10.3 mm in the frames with sawdust blocks, and at 4.8 mm in the frames with ordinary concrete blocks.

Table 10 shows the ductility values at the moment of failure in all frames analysed. The results show that blocks with wood additives provide greater ductility to structural systems, as it happened at the individual level with each block separately. Quantitatively, the static nonlinear analysis determines that the frames built with block walls with wood additives increase their ductility up to 80% with respect to ordinary concrete for the case of 2-storey frame and 53% in the 5-storey frame in the plastic zone (yield point-first failure). In the second plastic branch, where there is no structural resistance and only displacement, materials with wood aggregates show a shorter plastic zone before rupture.

### 6.2. Dynamic Analysis-Time History

Dynamic analysis-Time History is usually used to predict the nonlinear inelastic response of a structure subjected to a seismic event. Obviously, the linear elastic response can also be modeled considering elastic elements and/or low levels of excitation. Interested readers can find a brief description of the method in [49,69]. In this work, the direct integration of the equations of the movement is carried out by means of the well-known Newmark method (1959). The seismic action modeling is performed by introducing accelerograms in the supports. The time period (Δ*t*) used in the analysis was 0.01 s and taken from the Lorca earthquake register in both directions (X, Y). The information registered during the Lorca earthquake indicates that the most catastrophic accelerations were in the North-South direction; for this reason, the dynamic analysis was carried out with the acceleration data. The dumping of the structure is represented by a Rayleigh model, in which the dumping factor is equal to 5%, an average value used in some studies carried out in recent years for this type of buildings. 

**Figure 10:**
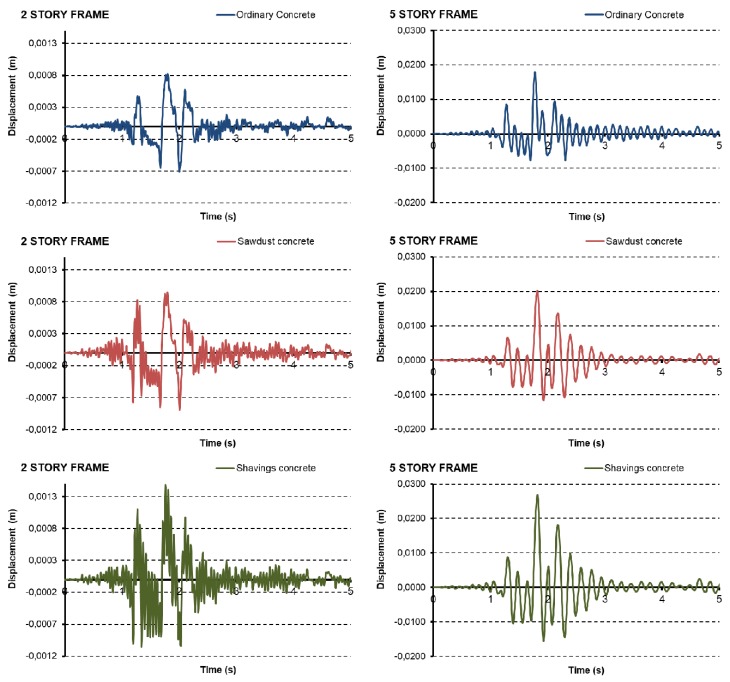
Frames time-history diagrams: (**left**) 2-storey frames; (**right**) 5-storey frames.

Figure 10 shows the seismic-resistant behaviour (Time-History diagrams) for all different types of frames and concrete blocks. Namely, the figures represent the top floor displacement of the frame time-history responses in Figure 6. The Time-History diagrams help obtain the maximum displacements for all types of structures (2- and 5-storey frames). For the 2-storey frames, an increase in maximum peak of displacements can be observed in the results with wood additives, from 0.82 mm for ordinary concrete to 0.95 mm and 1.5 mm for sawdust and wood shavings, respectively. Similarly, in the 5-storey frame, displacements increased from 18 mm for ordinary concrete to 20 mm and 27 mm for sawdust and wood shavings, respectively. 

According to Table 10 data and comparing the results obtained in the push over and dynamic analyses, it is possible to see that all displacements obtained in the 2-storey frames were in the elastic behaviour of the structure and they have not had any failure. On the other hand, in the 5-storey frame diagrams, it is noted that ordinary and wood shaving blocks achieve plasticity. Nevertheless, sawdust additive concrete blocks allow the structure to remain in the elastic zone. 

These results infer that the seismic behavior in structures with wood additives could be more flexible and suffer fewer fragile breaks, in accordance with that observed during the push over analysis, in particular for the sawdust case. Additionally, it is possible to conclude that buildings do not collapse in any case under the Lorca earthquake, evidencing the importance of having adequate seismic regulations.

## 7. Discussion

Prefabricated reinforced concrete blocks have achieved a wide diffusion in construction due to their remarkable strength, thermal and acoustic insulation, fire resistance and low humidity absorption. However, seismic countries such as Chile, Japan and many others also require materials with good seismic-resistant behaviour. In these countries, concrete blocks with greater ductility than ordinary concrete may be of great interest.

Taking into consideration the low density of wood in relation to its strength capacity, the main contribution of this work was to measure if some of the seismic-resistant properties of wood could be transferred to the concrete blocks, when used as an additive. 

The methodology followed included the experimental measurement of concrete properties such as water absorption and density in blocks made with ordinary concrete and blocks with different proportions of two types of wood additives. The compressive and flexural strengths were also measured. 

Throughout the study, the values registered were compared with the requirements established in the structural code regulations of Chile. This country was used as an example of a seismic country. In this sense, all materials used in this work comply with the requirements established by NCh148 cement (1969) [80], NCh163 aggregates (2013) [37], and NCh1498 water characteristics standards (1982) [81]. All the characteristics described in the Chilean Regulations meet the requirements established by other international regulations, such as the United States (ASTM 2004) [82], Mexico (NTC-M 2004) [83], Spain (CTE DEB SE F 2006) [52] or Eurocode 6 (2005) [84], all of them being less demanding than the Chilean regulations. 

It should also be noted that the minimum concrete structural strength in almost all the international regulations mentioned above is also lower than 12 MPa, the minimum required by Chilean Regulations. Similarly, the tested block dimensions complied with the minima established by the Chilean Concrete regulation for structural use NCh181 (1967). Particularly, 19 cm-thick blocks were used because they are the cheapest to build and are the most used in construction, due to the small size they take up in buildings in relation to interior distributions.

Regarding the density of the concretes used, they all remained above 2000 kg/m^3^; therefore, they could not be classified as lightweight concretes. However, it is obvious that the addition of sawdust or wood shavings decreased their density, with respect to ordinary concrete. Obviously, this density decrease is higher when a higher proportion of wood aggregate is added. However, for proportions of aggregates above 15%, the concretes no longer comply with the minimum structural strength regulations. In numerical terms, the substitution of 15% of gravel with 15% sawdust in the concrete manufacturing reduces its density by approximately 6%. Additionally, if we compare the two types of wood additives used, sawdust concretes reduce the weight of mixtures by about 6% more than that with added wood shavings.

As expected, the compressive strength of concrete blocks with sawdust or wood shaving additives also decreased as the wood/gravel proportion increased. However, despite this, all blocks with up to 15% of wood shavings or sawdust complied with the minimum strength requirements for structural use in Chile. The blocks’ flexural strength increases slightly when using wood additives. More precisely, the flexural strength in any of the blocks with wood additives is approximately 10% higher than that of traditional concrete. Comparing both wood additives, blocks with wood shavings were slightly more resistant, but the difference was less than 3% in all cases. 

Furthermore, the seismic-resistant behavior of frames built with blocks of up to 15% sawdust/wood shavings additives was studied through different static and dynamic analyses. From a seismic design point of view, one of the materials’ most important attributes is their ductility, which reflects the absorption capacity and energy dissipation that a structure can take before collapsing. In this sense, a building must dissipate the energy that the ground movement transfers to it during an earthquake. The most effective way to do this is by lateral deformation and local deterioration—internal damages where the energy transferred to the materials is converted into heat. If during a seismic episode, the horizontal deformation is not reached, the structure could collapse. Precisely, the maximum deformation that an earthquake demands on a structure is expressed through its ductility. 

The “non-linear” static behavior (push-over) of the analysed frames with wood additives also showed an improvement from the seismic-resistant point of view. These were less rigid and more resistant. The dynamic results (time-history) also confirmed a superior seismic-resistant behavior of the frames built with blocks with wood additive. Particularly, their greater flexibility meant that they suffered fewer fragile breaks in different elements of the frames. Additionally, the higher the frames, the more significant the performance improvement. 

Results of the maximum displacements and those from non-linear static calculations suggest that the buildings would have not collapsed under the Lorca earthquake, regardless of the type of blocks used. However, the maximum displacements of the frames were lower than the maximum displacements obtained from the “push-over” analyses. All the cases analysed remained within the elastic regime. The results obtained with the dynamic analyses, though, were not as conclusive as the static analyses. This is precisely because the dynamic analyses did not cause the structure to reach the plastic limit in any case (it did not suffer permanent deformations).

Finally, from an economic perspective, the introduction of wood shavings or sawdust in concrete instead of gravel does not significantly reduce the cost of the mixture. However, these wood composite concretes have structural benefits from a seismic point of view without increasing the cost of the structure. Additionally, the volumetric expansion that these types of concrete experience when adding wood-based materials could reduce their quantity demand. This may result in substantial economic savings in large-scale construction works. Depending on the amount of wood aggregate that the mixtures had, the expansion of these concretes could save up to 10–15% of the material.

## 8. Conclusions

This work studied how the partial replacement of gravel by sawdust or wood shavings in concrete mixtures can improve the seismic-resistant properties of concrete blocks. The methodology of the work included the construction of the blocks in the laboratory, the determination of their physical and resistant properties from construction and seismic points of view, as well as the comparison of their performance with ordinary concrete blocks when used in buildings with different storeys. The following conclusions can be drawn from the results:

- The compressive strength of ordinary concrete blocks is greater than that of blocks with wood additives. However, concretes with up to 15% wood additives still comply with the Chilean code NCh181 of concretes for structural use. In this regard, it is emphasized that Chile's seismic design standards are at a level of development similar to those offered by developed countries (such as Europe or the United States) in this field.
- The ductility of traditional concrete blocks is significantly lower than that of concrete with sawdust or wood shavings. A concrete with 15% wood shaving addition (in gravel weight) practically doubles the ductility of ordinary concrete, which considerably improves the seismic-resistant behavior of the block.
- Non-linear static push-over analyses in 2- and 5-storey frames also confirm a better structural performance in buildings with walls made of blocks with 15% sawdust or wood shavings. This behavior is due to the increase in ductility and flexural strength. This structural improvement is also more evident in the plastic branch of the capacity curves diagrams.
- The most ductile frames correspond to those with block walls, including wood shavings. These are 7% and 4.5% more ductile than the 2- and 5-storey frames built with block walls with sawdust aggregates, respectively. At the same time, these frames are 92% and 75% more ductile than the frames with ordinary concrete block walls.
- Dynamic analyses carried out using the North-South direction of the Lorca earthquake register do not reflect large differences in the use of different blocks. However, this is due to the fact that the design earthquake did not transfer enough intensity to bring the frames into their plastic regime.

Therefore, it can be concluded that buildings built with concrete blocks with up to 15% wood additives can greatly improve the seismic-resistant behaviour of buildings.

The present study is merely exploratory. A priori the blocks with wood shavings seem to exhibit better structural properties from a seismic point of view than the blocks with sawdust. However, the authors consider that these results may vary when taking into account other percentages of additives, types of wood, or wood granulometries. Additionally, other strengths of concrete and steel used, as well as other number of storeys and/or building configurations, should also be analysed to find optimal dosages applicable to a wider variety of contexts. As can be observed, the number of possible combinations to be checked is huge. Therefore, it is foreseeable that several additional studies will be necessary to cover them all.

## Figures and Tables

**Table 1:** Properties of a sample type of concrete manufactured for the study.

Property	Fine Aggregate	Coarse Aggregate	Mixture
Real density of dry saturated surface aggregate (kg/m^3^)	2618.6	2665.07	--
Density of dry aggregate (kg/m^3^)	2535.17	2635.29	--
Net density (kg/m^3^)	2766.59	2716.18	--
Water absorption (%)	3.29	1.13	--
Humidity (%)	4.5	2.0	--
Dry apparent density (kg/m^3^)	--	--	1455.5
Compacted apparent density (kg/m^3^)	--	--	1518.8
Content in holes (%)	--	--	42.36
Sponge (%)	--	--	44.76

**Table 2:** Dosages used in concrete blocks fabrication.

Material	Ordinary concrete	10% Sawdust or Shavings	15% Sawdust or Shavings	25% Sawdust or Shavings	40% Sawdust or Shavings
Water (%)	6.94	6.94	6.94	6.94	6.94
Cement (%)	17.04	17.04	17.04	17.04	17.04
Gravel (%)	37.15	33.43	31.58	27.86	22.29
Sand (%)	38.87	38.87	38.87	38.87	38.87
Additive (%)	-	3.73	5.57	9.29	14.86
Density (kg/m^3^)	2425	2283/2236	2247/2214	2185/2172	2084/2043

**Table 3:** Results of flexural tests in blocks.

Concrete Block Additives	Flexural (kg/cm^2^)
0%	3.2
10% Sawdust	3.5
15% Sawdust	3.8
10% Wood shavings	3.4
15% Wood shavings	3.6

**Table 4:** Deformations and ductility of all tested concrete blocks suitable for structural use.

Block Type	Plastic Deformation (δ_p_) (mm)	Ultimate Deformation (δ_u_) (mm)	Stiffness (kN/m)	Δ Stiffness (%)	Ductility (μ = δ_u_/δ_p_)	Δ Ductility (%)
Ordinary concrete	0.55	1.25	45.450	-	2.27	-
10% sawdust	0.47	1.54	34.040	−25.1	3.28	+44.5
10% shaving	0.46	1.62	32.610	−28.2	3.52	+55.1
15% sawdust	0.42	1.67	30.950	−31.9	3.98	+75.3
15% shaving	0.37	1.64	27.030	−40.5	4.43	+95.2

**Table 5:** Frame configurations.

	Height Frame (m)	Beams (cm^2^)	Columns (cm^2^)	Weight Frame (kN)	Fundamental Period (s)	Length Frame (m)
2-WA	7	20 × 35	20 × 30	358.70	0.232	21.5
2-WS	7	20 × 35	20 × 30	353.30	0.234	21.5
2-WW	7	20 × 35	20 × 30	340.96	0.234	21.5
5-WA	16	20 × 35	20 × 30	819.99	0.644	21.5
5-WS	16	20 × 35	20 × 30	807.55	0.642	21.5
5-WW	16	20 × 35	20 × 30	779.33	0.642	21.5

**Table 6:** Geometric and mechanic parameters obtained in the laboratory.

Parameter	Unit	Expression	Observations
Thickness panel	t (mm)		Lab data
Strut area	A_1_ (mm^2^)	A_1_ = b_w_ × t	According to [74]
Diagonal infill panel	d_w_ (mm)	d_w_	Lab data
Equivalent width of strut	b_w_ (mm)	b_w_ = 0.25 × d_w_	According to [73]
Elasticity block	E_b_ (N/mm^2^)		Lab data
Width block	b (mm)		Lab data
Mortar joint	j (mm)		Lab data
Elastic modulus	E_m_ (N/mm^2^)	$E_{m}=E_{b}\frac{\frac{b}{j}\;+\;1}{\frac{b}{j}\;+\;\frac{E_{b}}{E_{j}}}$	According to [74]
Height infill wall	h_w_ (mm)		Lab data
Mortar elasticity modulus	E_j_ (N/mm^2^)	Ej = 1000 × f_j_	According to [69,77]
Height storey frame	h (mm)		Lab data
Tensile strength	f_t_ (MPa)		Lab data
Compressive strength	f_m__*θ*_ (MPa)		Lab data
Maximum shear strength	*τ*_max_ (MPa)	*τ*_max_ = *τ*_0_ + μ f_m__*θ*_	According to [69]. *τ*_0 and_ µ are defined in Table 8
Density block	*γ*_p_ (KN/m3)		Lab data
Contact length	z (mm)	z = π/2λ	According to [73,78]
Dimensionless relative stiffness parameter	*λ*	$\lambda =\sqrt[{4}]{\frac{E_{m}tsin\theta }{4E_{c}I_{c}h_{w}}}$	According to [73,78]
Angle strut	*θ* (°)		Lab data
Inertia columns	I_c_ (mm^4^)	I = bh^3^/12	Lab data

**Table 7:** Numerical values for geometric and mechanic parameters obtained in the laboratory.

Unit	Ordinary Concrete	Sawdust Concrete	Shavings Concrete
t (mm)	191	191	191
A_1_ (mm^2^)	276970.21	276970.21	276970.21
d_w_ (mm)	5830.95	5830.95	5830.95
b_w_ (mm)	1457.74	1457.74	1457.74
E_b_ (N/mm^2^)	36000	17964.07	16463.41
b (mm)	192	192	192
j (mm)	20	20	20
E_m_ (N/mm^2^)	11709.30	4986.75	4429.08
h_w_ (mm)	3000	3000	3000
E_j_ (N/mm^2^)	37150.60	37150.60	37150.60
h (mm)	3400	3400	3400
f_t_ (MPa)	15	12	11.5
f_m__*θ*_ (MPa)	12.05	11.51	11.40
*τ*_max_ (MPa)	2.66	2.51	2.48
*γ*_p_ (KN/m3)	13.93	11.85	11.80
z (mm)	852.81	1055.68	1087.43
*λ*	0.00180	0.00149	0.00144
*θ* (°)	30.96	30.96	30.96
I_c_ (mm^4^)	675e6	675e6	675e6

**Table 8:** Geometric and mechanic parameters assumed from literature review.

Parameter	Units	Value	Observations
Friction coefficient	μ	0.3	According to [69]
Strain at maximum stress	*ε*_m_	0.0012	According to [74]
Ultimate strain	*ε*_u_	0.024	According to [74]
Closing strain	*ε*_d_	0.003	According to [74]
Strut area reduction strain	*ε*_1_	0.0006	According to [79]
Residual strut area strain	*ε*_2_	0.001	According to [79]
Bond shear strength	*τ*_0_ (MPa)	1.0	According to [79]
Percentage displacement X with respect to length	x_0i_ (%)	2.4	According to [48]
Percentage displacement Y with respect to height	y_0i_ (%)	10	According to [48]

**Table 9:** Empirical parameters.

Parameter	Suggested Values	Limit Values	Used Value (All Concretes)
α_s_	1.4–1.65		1.4
*γ*_un_	1.5–2.5	≥1	1.5
α_re_	0.2–0.4	≥0	0.2
α_ch_	0.3–0.6	0.1–0.7	0.7
*β*_a_	1.5–2.0	≥ 0	1.5
*β*_ch_	0.6–0.7	0.5–0.9	0.9
*γ*_plu_	0.5–0.7	0–1	1.0
*γ*_plr_	1.1–1.5	≥ 1	1.1
e_X1_	1.5–2.0	≥ 1	3
e_X2_	1.0–1.5	≥ 1	1.4
*γ*_s_	0.5–0.75		0.6

**Table 10:** Deformations and ductility of the frames.

Frame	Plastic Deformation (*δ_p_*) (mm)	Ultimate Deformation (*δ_u_*) (mm)	Ductility (*μ* = *δ_u_*/*δ_p_*)	Delta Ductility (%)
2-WA	10	25	2.50	-
2-WS	10	45	4.50	+ 80
2-WW	11	53	4.82	+93
5-WA	15	48	3.20	-
5-WS	21	103	4.90	+53
5-WW	22	115	5.23	+63

## Supplementary Materials

The following are available online at https://www.mdpi.com/1996-1944/12/9/1500/s1.
