# Supplementary material for: Mechanical Properties and Seismic Performance of Wood-Concrete Composite Blocks for Building Construction
Source: Materials (Basel). 2019 May 8;12(9):1500. doi: 10.3390/ma12091500 (PMC6539851; doi:10.3390/ma12091500)
Supplement: Supplementary file 1 [file materials-12-01500-s001.pdf]

materials

Supplementary Material

Description of the Chilean Regulations for Aggregates and Concretes

The Chilean standard initially proposes to perform different granulometry tests for aggregates and sand (fine aggregates) used in concrete. The granulometry tests include the conditioning, washing and drying in an oven of the aggregate sample as previous steps. After drying, the size of the granules of the aggregates is determined and homogenized by sieving.

Once the aggregates have been sieved, according to the recommendations of the standard, the density, humidity and water absorption of the fine aggregates (sand) is determined. These measurements are made with aggregate samples that passed through a 5 mm sieve. The sample is then immersed in water for 24 hours and allowed to dry until it reaches sufficient saturation so as to measure the mass of the superficially dried sample (m_{ss}). Subsequently, the sample is allowed to dry and stand for one hour at a temperature of 110°C, after which its mass (mm) is again measured hot. The sample is cooled and its mass measured definitively (m_s). Finally, the flask with water is weighed without the sample up to the calibration mark (m_a).

From the experimentally measured data, the real density of the superficially dried saturated aggregate (ρ_{Rss}), the density of the dried aggregate (ρ_{Rs}), the net density (ρ_N) and the water absorption (α) are calculated with the following expressions:

$$\rho_{Rss}\ (Kg/m^3) = \frac{m_{ss}}{m_a+m_{ss}-m_m} \cdot 1000 \quad (1)$$

$$\rho_{Rs}(Kg/m^3) = \frac{m_s}{m_a+m_{ss}-m_m} \cdot 1000 \quad (2)$$

$$\rho_N(Kg/m^3) = \frac{m_s}{m_a+m_s-m_m} \cdot 1000 \quad (3)$$

$$\alpha\ (\%) = \frac{m_{ss}-m_s}{m_s} \cdot 100 \quad (4)$$

Once the properties for fine aggregates have been determined, the process is repeated for coarse aggregates (gravels). Similarly, the gravel sample is prepared, washed and dried at a constant temperature of 110°C, cooled for 24 hours, submerged again and weighed in the submerged and suspended state *(A)*. Then it is weighed again saturated and superficially dry *(B)* and completely dry *(C)*. Then, the actual density of the superficially dried saturated aggregate (ρ_{RT}), the density of the dried aggregate (ρ_{Rs}), the net density (ρ_N) and the water absorption (α) are calculated according to:

$$\rho_{RT}(Kg/m^3) = \frac{B}{B-A} \cdot 1000 \quad (5)$$

$$\rho_{Rs}(Kg/m^3) = \frac{C}{B-A} \cdot 1000 \quad (6)$$

$$\rho_N(Kg/m^3) = \frac{C}{C-A} \cdot 1000 \quad (7)$$

$$\alpha\ (\%) = \frac{B-C}{C} \cdot 100 \quad (8)$$

Finally, both for fine aggregates and coarse aggregates, a sample of the aggregate is taken and its mass is recorded. The sample is dried in the oven at a constant temperature of 110°C and weighed again. The humidity H of the sample is calculated as (m_h-m_s)/m_s, where m_h is the mass of the wet sample and m_s is the mass of the dried sample.

Other important properties in the mixture characterization are the loose bulk density (ρ_{as}), the compacted bulk density (ρ_{ac}), the hollow content (h) and the foaming (E). These properties are determined by filling a container of volume (V) and mass (m_{rec}) known with the mixture without compacting (m_{s+rec}). Afterwards, the container is emptied and filled again, but now with a mixture compacted in three layers (m_{s+rec}). Once determined the volumetric and mass measurements, the densities of each mixture are obtained by:

$$\rho_{as}(Kg/m^3) = \frac{m_{s+rec} - m_{rec}}{V} \quad (9)$$

$$\rho_{ac}(Kg/m^3) = \frac{m_{c+rec} - m_{rec}}{V} \quad (10)$$

$$h = \frac{\rho_{Rs} - \rho_{ac}}{\rho_{Rs}} \quad (11)$$

$$E = \frac{\rho_{Rs} - \rho_{as}}{\rho_{Rs}} \quad (12)$$

Geometric Detail of the Formwork Used

Once the dosages were determined, the blocks were manufactured using a metal plate formwork (Figure 2) composed of three pieces:

Base of the container (42 x 23 cm), with holes in the perimeter and in the interior. Its function is to hold the pieces.

Piece composed of two truncated pyramids joined together by a metal bar. Its function is to provide shape to the interior holes of the block.

Side faces (19 x 39 cm). They allow the transport of the mold and the subsequent demolding of the piece.

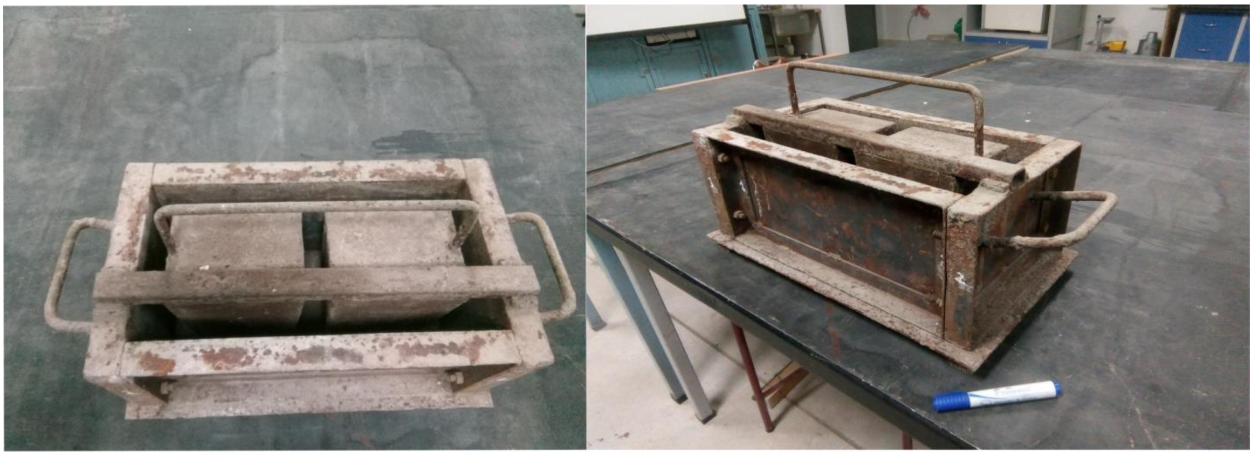

Figure S1. Formwork used in the construction of the blocks.
